# Localized Fetomaternal Hyperglycemia: Spatial and Kinetic Definition by Positron Emission Tomography

**DOI:** 10.1371/journal.pone.0012027

**Published:** 2010-08-06

**Authors:** Jianrong Yao, Chunlin Wang, Susan A. Walsh, Shanming Hu, Alexander B. Sawatzke, Diana Dang, Jeffrey L. Segar, Laura L. B. Ponto, John J. Sunderland, Andrew W. Norris

**Affiliations:** 1 Department of Pediatrics, University of Iowa Carver College of Medicine, Iowa City, Iowa, United States of America; 2 Small Animal Imaging Core, University of Iowa Carver College of Medicine, Iowa City, Iowa, United States of America; 3 Department of Radiology, University of Iowa Carver College of Medicine, Iowa City, Iowa, United States of America; University of Padova, Medical School, Italy

## Abstract

**Background:**

Complex but common maternal diseases such as diabetes and obesity contribute to adverse fetal outcomes. Understanding of the mechanisms involved is hampered by difficulty in isolating individual elements of complex maternal states *in vivo*. We approached this problem in the context of maternal diabetes and sought an approach to expose the developing fetus *in vivo* to isolated hyperglycemia in the pregnant rat.

**Methodology and Principal Findings:**

We hypothesized that glucose infused into the arterial supply of one uterine horn would more highly expose fetuses in the ipsilateral versus contralateral uterine horn. To test this, the glucose tracer [^18^F]fluorodeoxyglucose (FDG) was infused via the left uterine artery. Regional glucose uptake into maternal tissues and fetuses was quantified using positron emission tomography (PET). Upon infusion, FDG accumulation began in the left-sided placentae, subsequently spreading to the fetuses. Over two hours after completion of the infusion, FDG accumulation was significantly greater in left compared to right uterine horn fetuses, favoring the left by 1.9±0.1 and 2.8±0.3 fold under fasted and hyperinsulinemic conditions (*p*<10^−11^ n = 32-35 and *p*<10^−12^ n = 27–45) respectively. By contrast, centrally administered [^3^H]-2-deoxyglucose accumulated equally between the fetuses of the two uterine horns. Induction of significant hyperglycemia (10^3^ mg/dL) localized to the left uterine artery was sustained for at least 48 hours while maternal euglycemia was maintained.

**Conclusions and Significance:**

This approach exposes selected fetuses to localized hyperglycemia *in vivo*, minimizing exposure of the mother and thus secondary effects. Additionally, a set of less exposed internal control fetuses are maintained for comparison, allowing direct study of the *in vivo* fetal effects of isolated hyperglycemia. Broadly, this approach can be extended to study a variety of maternal-sided perturbations suspected to directly affect fetal health.

## Introduction

The fetus normally is maintained in a protected environment, sheltered by maternal and placental homeostasis and detoxification mechanisms. When these protections fail or are breached, significant health consequences to the fetus can occur, inducing malformations [1], neonatal maladies [2], and/or disease susceptibilities that may not manifest for decades [3].

In simple cases, fetal consequences are induced by direct action of a single inciting agent on the fetus or non-maternal extra-fetal tissues [4]. By contrast, in complex cases, the maternal state consists of compound perturbations, such that it is often unclear which specific perturbations of the fetal environment are responsible for the adverse fetal outcomes [5], [6]. Examples include diabetes mellitus [7] and obesity [8]. In these complex phenomena, potential mediators of fetal damage may include alterations in numerous circulating micro- and macro- nutrients [9], inflammatory agents [10], and reactive oxygen species [11]. It often is difficult to induce isolated single maternal perturbations related to the above complex phenomena and *in vivo* model approaches which dissect specific maternal-sided contributors to fetal pathology are needed.

We have approached this problem in the context of fetal diabetes exposure, which recapitulates many of the above characteristics. Diabetes exposure during early fetal development leads to birth defect risk [7] whereas exposure later in gestation leads to neonatal maladies [2] as well as risk of insulin resistance and type 2 diabetes later in life [12], [13]. The Freinkel hypothesis of fuel-mediated teratogenesis posits that the excess exposure to metabolic fuels increased by maternal diabetes leads to the adverse fetal and offspring outcomes [9]. Studies using embryo culture [14], [15] and epidemiologic correlation [16] have lent credence to this hypothesis, as have fuel infusions sufficient to induce systemic maternal elevations [17] though the latter are complicated by metabolic spillover [18] such as insulin resistance due to lipid infusion or hyperlipidemia due to glucose infusion [19]. The overall conclusion from these studies is that although hyperglycemia is likely the primary fuel that incites fetal pathology [20] additional fuels contribute as well, including ketones, fatty acids, triglycerides, and branched chain amino acids [21]-[23]. However, direct determination of the effects of individual fuels *in vivo* on fetal development and offspring health has been difficult to achieve.

To further address this need, it is hypothesized that glucose infused into the arterial supply of the uterus would highly expose fetuses to hyperglycemia while minimizing maternal exposure and secondary effects. We took advantage of the bicornate nature of the rat uterus, wherein embryos develop in either the left or the right horns of the uterus. This is of great experimental utility, since the two horns of the uterus have distinct blood supplies [24]. Because circulating glucose is rapidly cleared from plasma [25], the amount of infused glucose reaching the central circulation of the mother and thus the contralateral fetuses should be significantly less than the amount reaching the fetuses on the side of the infusion. Here, we validate this approach, testing for differential uterine horn hyperglycemic exposure using positron emission tomography (PET) imaging and glucose metabolic tracers to define the temporal and spatial disposition of locally infused glucose.

## Results

### Uterine artery catheter

**Figure 1:**
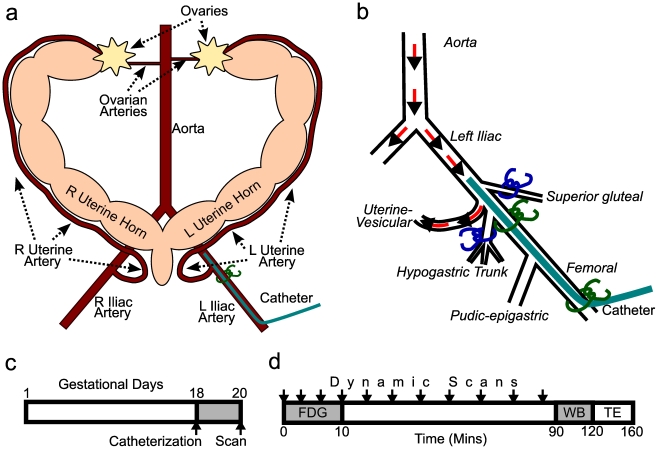
A model for selective fetal exposure. (**a**) The vast majority of blood flow to each uterine horn is supplied by each horn's uterine artery [24], which derives from the iliac artery. Therefore, infusate from a catheter appropriately placed in the left iliac artery should experience first pass exposure to fetuses in the left, but not right, uterine horn. The fetuses in the right uterine horn thus serve as internal controls. (**b**) In surgical approach “A”, ligatures (green) were tied around the left internal iliac artery just inferior to the uterine artery take off and around the femoral artery. Surgical approach “B” included additional ties (blue) around the left superior gluteal and hypogastric trunk arteries. (**c**) Experimental time-line. (**d**) Dynamic PET of the abdomen was initiated upon infusion of FDG, followed by a whole-body scan (WB) at 90-120 minutes, transmission and emission contamination scans (TE) at 120-160 minutes, and tissue collection after euthanasia at 160 minutes.

Blood flow to each uterine horn is primarily provided by its uterine artery, reaching flow rates of 1-2 mL/min near the end of gestation [24], [26]. An infusion catheter was advanced from the left femoral artery such that the tip lay just upstream of the left uterine artery takeoff (Fig. 1a). Competing blood flow was reduced by either simply ligating the iliac artery just downstream of the uterine artery takeoff (surgical approach “A”) or by, more extensively, also tying off two nearby competing arteries (approach “B”) (Fig. 1b). Catheter placement was performed on gestational day #18 and rats were studied by PET scan on gestational day #20 (Fig. 1c). Maternal weight increased pre-surgery to pre-scan from 323±4 to 330±4g (*p*<0.05, n = 11).

### Disposition of infused glucose tracer

**Figure 2:**
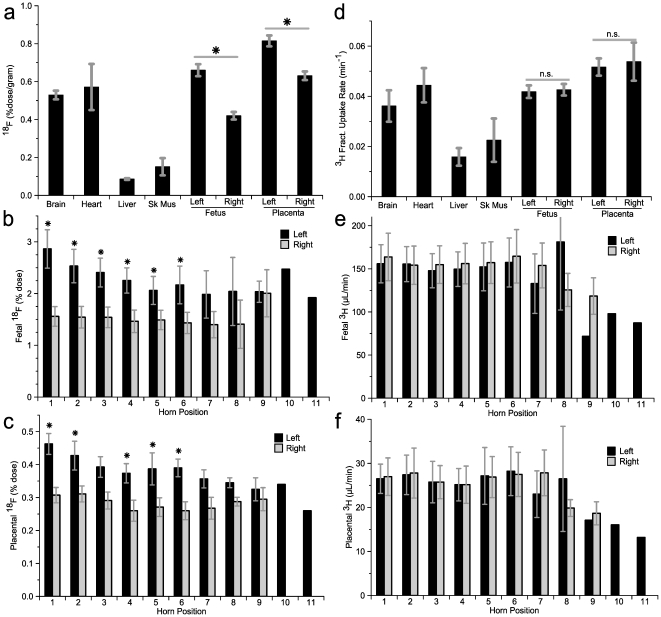
Final accumulation of glucose tracers. (**a**) Per gram accumulation of ^18^F in various tissues at 160 minutes after FDG infusion. (* *p*<10^−5^ for left versus right, n = 76–79). Per fetus (**b**) and per placenta (**c**) accumulation of ^18^F, based on uterine horn position. The fetus/placenta closed to the cervix is counted as position 1. * *p*<0.05 left versus right. (**d**) Clearance of ^3^H into various tissues as measured after completion of the scan (n.s. not significant for left versus right). Per fetus (**e**) and per placenta (**f**) accumulation of ^3^H, based on uterine horn position.

We hypothesized that glucose infused via the left uterine artery catheter would have a greater chance of being taken up by the fetuses in the left uterine horn as compared to those in the right uterine horn. To test this directly, we used the glucose tracer [^18^F]fluorodeoxyglucose (FDG) and positron emission tomography (PET). An 8.9±1.1 min bolus infusion of FDG was initiated and the animals were scanned for the ensuing 120 minutes (Fig. 1d). Serum ^18^F concentration peaked at 20 minutes, showing good correlation between samples collected from the tail or right femoral artery (Fig. S1a,b). Serum glucose levels remained euglycemic (132±2 mg/dL, n = 226) and unchanged throughout the scan (Fig. S1c,d). The ^18^F content of tissues, fetuses and placentae were determined after euthanasia at 161±3 in. As expected, brain and heart showed high degrees of FDG accumulation, while liver and skeletal muscle experienced less (Fig. 2a). Like heart and brain, right-sided fetuses and placentae were exposed to FDG only after it entered the systemic circulation. The right-sided fetuses showed intermediate avidity for FDG, accumulating FDG levels nearer that of brain than of muscle, whereas right-sided placentae showed avidity for FDG that rivaled that of brain. Importantly, left-sided fetuses and placentae accumulated significantly more FDG than their right-sided counterparts (Fig. 2a). The uterine horn positions closest to the cervix showed the greatest differential uptake (Fig. 2b,c), though the left-greater-than-right pattern was present at nearly all positions. On average, each left-sided fetus accumulated 2.3±0.1% of the total administered dose whereas each right-sided fetus accumulated only 1.5±0.1% (*p*<10^-8^ for left versus right, n = 76-79).

### Disposition of centrally administered glucose tracer

As a control for the unilaterally infused FDG, we centrally administered [^3^H]-2-deoxyglucose (2DG), a similar glucose tracer, via subcutaneous injection just prior to initiation of the PET scans. The resultant ^3^H serum activity exhibited a similar profile to that of ^18^F (Fig. S1e,f). As with FDG, brain and heart showed greater avidity for 2DG than did liver and skeletal muscle (Fig. 2d). Fetuses and placentae also showed great avidity for 2DG. However, in contrast to ^18^F, the accumulation of ^3^H was symmetric between the fetuses and placentae from the two uterine horns (Fig. 2d). This was true across all uterine horn positions (Fig. 2e,f).

### Dynamic PET imaging

**Figure 3:**
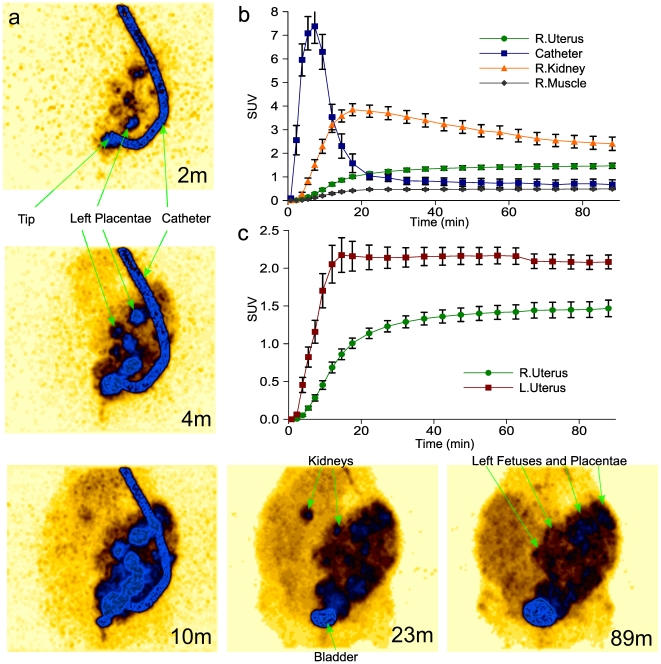
Dynamic PET imaging. (**a**) Representative dynamic PET images, shown as maximum intensity projections. Dynamic scans were centered on the abdomen. Minutes (“m”) after initiation of infusion are shown on each image. (**b**) SUV evolution for several anatomic structures. (**c**) SUV evolution in the left and right uterine horns.

Dynamic PET images were acquired for the first 90 minutes after initiating FDG infusion, with the field of view encompassing the abdomen. By several minutes, the infusion catheter was clearly visible (Fig. 3a). Early frames showed accumulation of FDG in discoid structures superior to the catheter tip, the location and size of the accumulation being consistent with the placentae of the left uterine horn. Over the ensuing frames, the entire left uterine horn regions became progressively more uniformly populated with radioactivity, representing movement of FDG into fetal tissues as well. As expected, the kidneys became symmetrically visible and then faded as FDG accumulated in the urinary bladder (Video S1).

These visualized phenomena were recapitulated in mean standardized uptake values (SUVs) measured for these regions of interest (Fig. 3b), reinforcing the qualitative impressions gleaned from visualization of the dynamic images. The infusion catheter SUV peaked early and intensely, fading significantly by 20 minutes. Renal SUV peaked at about 20 minutes, whereas right uterus SUV reached a near plateau by 90 minutes, with far lower SUV in skeletal muscle. Importantly, the left uterine horn accumulated more FDG than the right uterine horn (Fig. 3c), primarily due to enhanced FDG accumulation during the catheter infusion phase, consistent with greater first pass exposure to glucose tracer.

### Whole-body PET imaging

**Figure 4:**
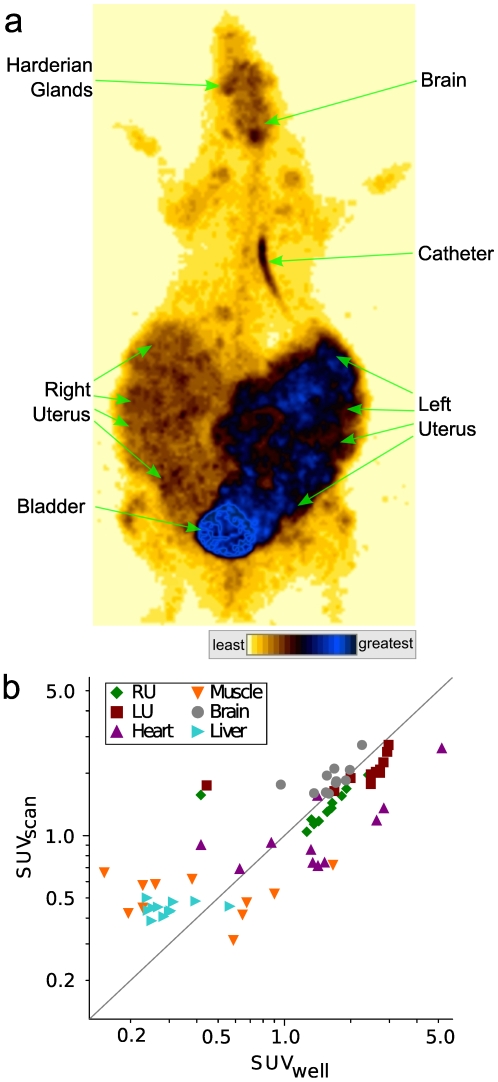
PET whole-body imaging. (**a**) Representative maximum intensity projection whole-body image. Color-intensity gradations are indicated from least to greatest. (**b**) Correlation between standardized uptake values (SUV) for volumes of interest as measured from the final whole body image (SUV*_scan_*) and for the corresponding tissues as determined by gamma well counter measurement, dose administered, and weight of the rat and the tissue (SUV*_well_*) (*r* = 0.82, n = 66, *p*<10^-16^). Abbreviations: RU – right uterine horn; LU – left uterine horn.

A whole-body PET image was acquired at 90-120 minutes after FDG infusion (Fig. 4a). High accumulations of FDG were evident in glucose-avid tissues including the brain, harderian glands and variably the heart and thoracic aspect of the catheter. As expected, FDG concentrations were high within the bladder. The uterine horns also were dominant structures of FDG accumulation, with higher concentrations in the left uterine horn. There was good correlation between SUVs determined either from the whole-body scan for selected volumes of interest (VOIs) or from direct well-counter-based assay of the ^18^F content of the corresponding tissues collected after the scan (Fig. 4b), confirming the above qualitative image-based impressions. Despite the overall excellent correlation between *in vivo* imaging and *ex vivo* radioactivity concentration determinations (Fig. 4b), minor discrepant trends were apparent. In particular, PET-based SUV tended to underestimate uterine and heart SUV. This likely is due to inactive uterine structures (e.g. amniotic fluid) included in the image-based VOI analyses but not in the radioactivity concentration determinations of extracted tissue such as the placentae and fetuses which were blotted dry prior to weighing and counting. The same may apply to the heart, where the image-based VOI analyses would include the blood in the ventricles that would not be included in the weights and well counter-based assays. In contrast, PET-based SUV of brain tended to be higher than well-counter SUV, perhaps due to bias in drawing brain regions on “hotter” portions of the images.

### Fetal response to catheter

Ligation of the uterine artery leads to intrauterine growth retardation [27]. Gestational days 18-20 are a time of tremendous growth; for example, one rat strain experiences a nearly tripling of fetal weight during this interval [28]. It is thus conceivable that our uterine artery catheter approach might impede blood flow to the uterus thus impairing fetal growth. Arguing against this possibility, there was no difference in fetal weight between the left and right uterine horns at 3.52±0.06 versus 3.54±0.06 g (*p* = 0.87, n = 76-79). There was a modest increase in placental size in the left compared to right uterine horns at 0.50±0.01 versus 0.46±0.01 g (*p*<0.005, n = 76-79). Comparing by uterine position, no differences between left and right fetuses were apparent, whereas the slightly increased placental size on the left was observed in several positions (Fig. S2). Fetuses and placentae which developed further from the cervix tended to be smaller than those more proximal to the cervix, a trend which was observed both on the right and left sides. No fetal deaths or resorptions were observed among these scans and there was no difference in the number of fetuses between the left and right uterine horns (left 6.9±0.6 fetuses, right 7.2±0.4 fetuses, *p* = ns, N = 11).

### Uterine artery catheter drainage

**Figure 5:**
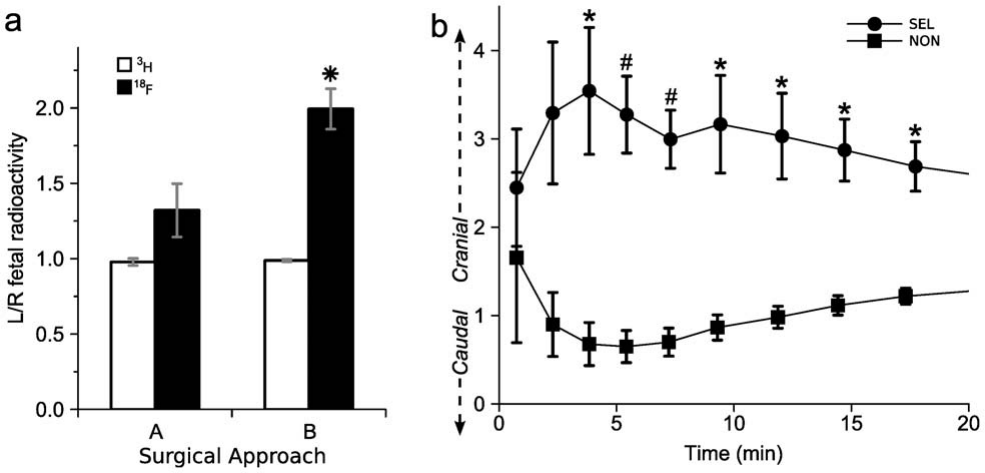
Impact of early FDG pattern on differential glucose exposure. (**a**) Impact of surgical approach on differential ^18^F (black bars) but not ^3^H (white bars) accumulation (* *p*<0.05 for difference between approaches, n = 5–6). (**b**) The ratio of ^18^F cranial versus caudal to the catheter tip is plotted over time for scans that exhibited a final left/right uterine horn ^18^F ratio of >1.6 (SEL) or <1.6 (NON). (*p<0.01, #p<0.001, n = 5–6).

Some scans exhibited a lack of differential FDG accumulation between the left and right uterine horn structures. We noted that the scans lacking differential uptake typically also demonstrated a predominance of early FDG distribution into non-uterine, caudal structures such as the tail or left hindquarter (Fig. S3). We reasoned that this was likely due to competing drainage of infusate into other nearby arterial trunks arising from the iliac artery. By dissection, we identified the hypogastric trunk and superior gluteal arteries as potential diverting arterial drainages (Fig. 1b). We thus tested the hypothesis that surgical approach “B”, in which we additionally tied off these two arterial trunks, would result in a more differential FDG accumulation between the two uterine horns. There was left-dominant FDG accumulation in both surgical approaches, however surgical approach “B” as compared to approach “A” resulted in greater left-sided selectivity calculated either per mother (Fig. 5a) or per fetus (left/right = 1.9±0.1 versus 1.3±0.1, n = 27-30,38 for “B” and “A” respectively). By contrast, the ratio of 2DG accumulation between the left and right horns was essentially 1.0 in both surgical approaches.

We further tested the early FDG infusate drainage pattern for association with the eventual left-versus-right uterine FDG accumulation. We divided the scans into those that showed strong or weak left-versus-right differential FDG accumulation on the 90 minute PET image. For each animal, we defined two volumes of interest, one cranial and one caudal to the infusion catheter tip, representing flow into the left uterine region or into caudal structures of the rump, tail and hindquarter. The animals with eventual strong left-versus-right differential accumulation exhibited increasing cranially-directed FDG peaking at 5 minutes, indicative of immediate flow of the catheter infusate into the left uterine structures (Fig. 5b). By contrast, among animals exhibiting eventual weak left-versus-right differential accumulation, the pattern was reversed, with a caudally-directed inverted peak at 5 minutes, indicative of direct infusate flow not towards the uterine horn but rather into inferior structures. Thus, in a portion of our scans, the infusate failed to experience first-pass exposure to the left uterine horn; a situation improved by surgical approach “B”.

### Optimizing differential exposure

**Figure 6:**
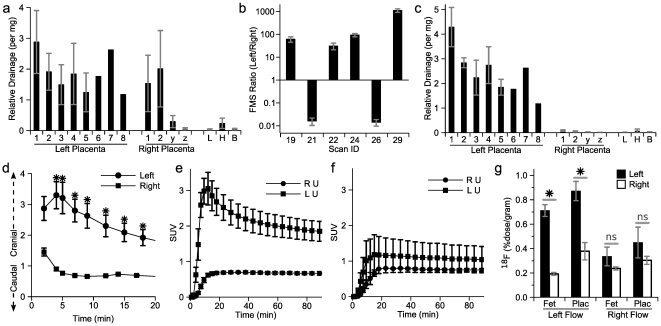
Impact of infusate flow on differential glucose exposure. (**a**) Accumulation of fluorescent microspheres (FMS) in placenta by laterality and position (1 is closest to cervix; z is closest to ovary) and tissues (L, liver; H, heart; B, brain). The number of FMS normalized to weight and total number, such that a tissue receiving an average number per gram would have a relative drainage of 1. (**b**) Relative accumulation of FMS between left and right, dividing the average number per left placenta by the average number per right placenta, among six individual scans. (**c**) Accumulation of FMS in placenta and tissues only among scans showing left-dominant FMS accumulation. (**d**) The ratio of FDG concentration cranial versus caudal to the catheter tip for scans that exhibited a left- or right-dominant FMS pattern (*p<0.05, n = 2–4). (**e-f**) SUV in left uterine (LU) versus right uterine (RU) horns for (**e**) left-dominant or (**f**) right-dominant FMS patterns. (**g**) Final accumulation of FDG in left and right fetuses and placenta in scans showing left- or right- dominant FMS accumulation. (*p<10^-4^, for left versus right ^18^F, n = 19–25; ns *p* = not significant, n = 8–20).

To explore the above considerations and optimize differential exposure, we performed an additional series of PET scans in which we infused 15 µm fluorescent microspheres (FMS) via the left uterine artery catheter at the conclusion of the scan. The FMS are unable to traverse the arterial to venous circulation, and thus are used to quantitate blood flow to tissues including placenta [29]. As expected, far greater FMS accumulated in placentae of the left uterine horn than in structures not drained by the left uterine artery including heart, liver and brain (Fig. 6a). However, the first two right-sided fetuses also received a large portion of FMS on average, though the terminal two right-sided fetuses did not. Interestingly, the ratio of left-versus-right FMS was extremely discrepant from scan to scan, with most scans showing a strong left-sided selectivity but a few scans showed a right-sided selectivity paradoxically (Fig. 6b). The change in selectivity was due primarily to a reduction in left-sided (500±80-fold, *p*<0.0005, n = 8-19) and less so to an increase in right-sided (8±3-fold, *p*<0.005, n = 8-15) FMS accumulation. The reasons for this alteration in flow pattern were not readily discerned on dissection. When these right-sided-FMS-flow scans were excluded, the remaining animals showed a very high degree of left-sided flow selectivity (Fig. 6c). As hypothesized, the right-sided-FMS-flow scans exhibited a caudally-directed pattern of FDG at 5 minutes as compared to the strongly cranially-directed FDG appearance in the left-sided-FMS-flow scans (Fig. 6d). Thus, a portion of catheterizations were unsuccessful in directing infusate flow to the left uterus. Importantly, across all scans, the chances of a selective infusion, as defined as a fetal left-versus-right FDG selectivity ratio >1.6, were far greater with surgical approach “B” (10 of 11) than “A” (2 of 6).

To further enhance the degree of left-versus-right uterine horn FDG exposure, these scans (Fig. 6) were performed under hyperinsulinemic conditions during euglycemic clamp. We reasoned that, since insulin action shortens the plasma half-life of circulating glucose, this would reduce the relative amount of left uterine artery infused FDG reaching the right uterine horn. Indeed, uterine horn SUVs were much stronger on the left than on the right among the animals with left-sided-FMS-flow (Fig. 6e), a pattern not seen in right-sided-FMS-flow animals despite hyperinsulinemia (Fig. 6f). Furthermore, the right-sided-FMS-flow animals showed a lack of differential FDG accumulation between the left and right uterine horns (Fig. 6g, right-flow group). By selecting left-sided-FMS-flow animals only under hyperinsulinemic conditions, there was a 3.7-fold increased accumulation of FDG in the left- versus right- uterine horn fetuses (*p*<10^-4^, n = 19-25) (Fig. 6g, left-flow group; this data is shown by uterine horn position in Fig. S4).

#### Chronic glucose infusions

**Figure 7:**
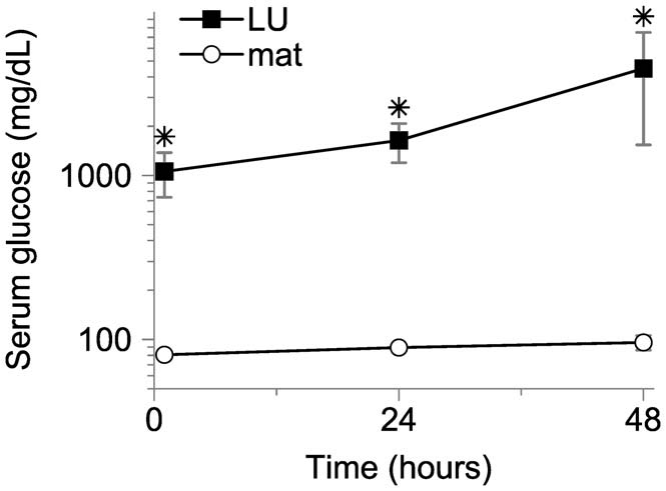
Chronic glucose infusion. Glucose was infused on gestational days #18-20 at 4 mg/min via the left artery catheter. Serum was sampled downstream of the infusion catheter tip (LU, black squares) and from the maternal tail tip (mat, open circles). *p<0.05.

We assessed the ability of this approach to maintain differential glucose exposure between the left uterine artery and systemic maternal circulation. A double lumen catheter was placed on gestational day #18 using surgical approach “B”. Glucose was infused for 48 hours via the upstream port at 4 mg/min and blood sampled via the downstream port. This produced sustained and severe localized hyperglycemia at the left uterine artery takeoff, while normal glycemia was maintained in the maternal systemic circulation (Fig. 7).

## Discussion

Here, we quantify an approach to expose the fetus *in vivo* to isolated hyperglycemia by infusion of glucose into one of the unilateral uterine arteries. As hypothesized, the ipsilateral fetuses were exposed to significantly more hyperglycemia than contralateral fetuses, with substantive first pass clearance of glucose into the ipsilateral placentae and fetuses.

To best take advantage of this approach, ideally all of the catheter infusate would be directed into uterine artery flow. Although this could be achieved by direct catheterization of the distal uterine artery, this would likely impede uterine blood flow. We used a surgically simpler approach, similar to that reported in studies of maternal-fetal metabolite [30] and toxin [31] transfer, placing the catheter tip in the iliac artery near the uterine artery takeoff [30]. Because several nearby arteries can divert a portion of the infusate to structures other than the uterus, there are competing interests between ligating arteries that divert flow and minimizing surgical dissection. In our study, ligating two nearby arteries improved uterine horn selectivity with a minimum of additional dissection. Although additional ties could be placed, we believe our approach “B” represents a reasonable balance between selectivity and surgical intervention. In some animals, catheter flow to the left uterine horn was unexpectedly negligible as assessed by early images from PET scanning, corroborated by fluorescent microspheres (FMS). Importantly, this result illustrates that FMS can be used as a means to assess catheter drainage without the need for PET. FMS could be used to select those animals which exhibit the desired flow pattern, thus removing from consideration infusions that otherwise would dilute the effect under study. On the other hand, strictly standardizing by FMS flow could reduce the within-experiment variation in unintended ways, reducing the degree to which results could be generalized [32]. It is important to note that selection by flow pattern was not required to obtain statistically significant degrees of differential uterine horn exposure even when averaging across a small number of animals.

The approach of directly providing excess macronutrients *in vivo* to fetuses in one horn of the uterus has several advantages over systemic induction of hyperglycemia [33] or diabetes. Importantly, the induced hyperglycemia is localized. For example, infusion of 9 and 15 mg/min glucose induces mild and marked systemic hyperglycemia in pregnant rats [34], whereas we find that infusion of 4 mg/min glucose into the uterine artery at term raises the local glucose concentration markedly but has little effect on maternal systemic glucose levels. Another advantage is that the contralateral fetuses serve as an ideal internal control. Fetuses from both sides are exposed to essentially the same maternal milieu, with the exception that the left-sided fetuses are additionally exposed to the localized hyperglycemia. This approach can be thought of as an *in vivo* compliment to *in vitro* embryo culture, which has been very helpful in determining the effects of metabolic fuel excesses on fetal development but does not perfectly recapitulate the *in vivo* situation [33], [35], [36]. In fact, the idea of providing excess macronutrients *in vivo* to fetuses in one horn of the uterus is not new, having been explored via transamniotic feeding [37] for example. One additional advantage of directly infusing glucose into one uterine artery is that the timing of hyperglycemia exposure can be finely controlled temporally. For example, the fetal effects of brief repeated episodes of hyperglycemia, such as occur during pregnancy complicated by glucose variability in unstable type 1 diabetes, could be tested.

This approach has several limitations. As implied above, this approach does not recapitulate the full effects of diabetes during pregnancy, but rather is limited to the effects of hyperglycemia local to the fetus. However, this limitation is of value to help identify the individual metabolic fuels that contribute to fetal risk. It is important to know which maternal metabolic fuels elevated in diabetes to target in order to reduce fetal pathology [38], especially given that maternal-sided interventions might have advantages over fetal-sided interventions [39]. Additionally, the present work was limited to late gestation, during which diabetes adversely affects fetal growth, neonatal health [2], and programming of adult disease [12], [13] but does not cause teratogenesis [7]. To adapt this technique to the study of diabetes-related birth defects, the catheter would need to be placed earlier in gestation to allow hyperglycemic infusion during fetal organogenesis.

Our immediate plans with this model are to study the effects of localized hyperglycemia on placental blood flow, fetal growth, and islet development late in gestation. We also note that this approach could be applied to other agents beyond glucose. A general requirement for utility in this approach is that the agent tested needs to be cleared rapidly from the circulation, otherwise significant amounts will reach the contralateral horn. The half-life of glucose in the circulation of the rat is on the order of 10-40 minutes [25]. This technique may be well suited to study other rapidly cleared diabetes-related metabolites such as fatty acids [40], triglycerides [41], and ketones [42]. Thus, this model is ideally suited to test components of the Freinkel hypothesis *in vivo*. Additionally, this method is likely applicable to other categories of agents that affect mother and fetus, such as various toxins, pharmaceuticals, and reactive oxygen species. We conclude that this model approach creates isolated hyperglycemic fetal exposure while maintaining relatively unexposed control fetuses in the opposing uterine horn and that this approach is likely well-suited to study of other agents that may impact on fetal health.

## Materials and Methods

### Animals

All procedures were performed within the regulations of the Animal Welfare Act and the National Institutes of Health Guide for the Care and Use of Laboratory Animals, and were approved by the Institutional Animal Care and Use Committee of the University of Iowa. Timed, pregnant Hsd:Sprague Dawley SD rats (Harlan Laboratories, Inc.) were allowed one week to acclimate. Gestational day 0 was defined as the day of initial vaginal plug detection.

### Uterine artery catheter

On gestational day 18, a vascular catheter draining into the left-sided uterine artery was placed as follows. Anesthesia was induced and maintained with inhalational isoflurane mixed with oxygen. The femoral triangle was opened, and a stretched, sterilized PE10 catheter (Instech Laboratories Inc., Plymouth Meeting, PA) was inserted and secured 1.65 cm retrograde into the femoral artery, thus placing the tip several mm superior to the uterine artery takeoff from the common iliac. The left inferior peritoneal space was then opened and a silk ligature tied around the iliac artery inferior to the uterine artery takeoff, typically just inferior to the hypogastric trunk artery. In selected surgeries, the superior gluteal and hypogastric trunk arteries were identified [43] and tied with silk ligature at their origin. A right sided femoral artery catheter was likewise placed except without intraperitoneal procedure. Catheters were tunneled subcutaneously to a mid-scapular exit and connected to a dual channel infusion swivel (Instech) allowing the rat freedom of movement. Postoperative analgesia included bupivacaine and buprenorphine. Saline with 5 U/mL heparin was infused at 10 µl/min to maintain each catheter's patency.

### PET imaging

On gestational day 20, rats were anesthetized with 1.5% isoflurane after overnight fast (13.6±0.9 h). Each animal was maintained on a heated bed in dorsal recumbency, blood glucose measured, a bladder catheter and rectal temperature probe placed, and positioned in a MOSAIC microPET scanner (Philips Medical Systems, Milpitas, CA, USA) with the abdomen completely within the field of view as confirmed by transmission scan using ^137^Cs. FDG was delivered in its standard buffer, containing a trivial amount of glucose (<∼6 mg/dL, formed as by-product of the FDG synthesis), used for human clinical infusions. At time zero, a dose of 33±2 MBq ^18^FDG in approximately 175 µL was infused at 20 µL/min via the uterine artery catheter. Dynamic PET imaging (120 transaxial slices, 1 mm slice thickness) and blood sampling commenced at the start of the FDG infusion (t = 0). At 90-120 minutes, a whole-body image of the rat from lower extremities to head was acquired (230 transaxial slices, 1 mm slice thickness) by movement of the scanner bed through the gantry. The rat and its fetuses were then euthanized with 150 mg/kg intravenous pentobarbital and the fetuses and placentae collected by Caesarean section. The native positioning of the left and right-sided fetuses was photodocumented prior to removal. Plasma samples and excised tissues were weighed and the ^18^F content determined by assay in a previously calibrated gamma NaI well-counter with decay correction to the start of the radiotracer infusion. 

### Quantitative image analysis

Transaxial slices were reconstructed using a 3D Row Action Maximum Likelihood Algorithm (Philips Medical Systems) on projection data corrected for random coincidences, scatter, and dead time. All images were attenuation- and decay- corrected. PET images were scaled in standardized uptake values (SUV) by normalizing to the administered FDG dose and the weight of the animal. Image analysis was performed using *PMOD 3.0* (PMOD Technologies Ltd., Zurich, Switzerland). VOIs were defined manually on transverse sections while viewing SUV data displayed in four dimensions (transverse, sagittal, and coronal planes and time). The anatomic regions representing the left and right uterine horns were defined while comparing PET images to the respective photodocuments.

### Central 2-deoxyglucose tracing

In selected animals, 80 µCi of 2-[1,2-^3^H(N)]-deoxy-D-glucose (2DG) (New England Nuclear, PerkinElmer, Waltham, MA) was injected subcutaneously in the axilla immediately before initiating ^18^FDG infusion. Tritium content was determined in tissue and plasma samples after ^18^F decay. Tissues were dissolved in 4N KOH overnight, the supernatant neutralized, and ^3^H content determined by liquid scintillation counting. Glucose concentrations were measured on whole-blood or plasma with a One Touch Ultra meter (LifeScan Inc., Milpitas, CA).

### Fluorescent microspheres

At the conclusion of selected PET scans, 1×10^5^ fluorescent microspheres (FMS) (polystyrene, 15 µm, # F-8844, Invitrogen Corp., Carlsbad, CA) were infused at 20 µL/min via the uterine artery catheter. After ^18^F decay, the FMS content of selected tissues was determined as follows. Each tissue was dissolved in 2.5 mL/g-tissue 4N KOH:2% Tween 80 overnight, then FMS collected by filtration through a 5 µm pore size polycarbonate membrane. After rinsing with 2% Tween 80, FMS were dissolved from the membrane in 1 ml 2-ethoxyethyl acetate. Fluorescence was quantitated in polypropylene multiwell plates using excitation:emission frequencies of 492∶520 nm and compared to a standardized curve.

### Euglycemic hyperinsulinemic clamp during PET

After induction of anesthesia, insulin was infused via the right femoral vein at 20 mU/kg/min. Glucose was infused via the same catheter to maintain blood glucose at 90 mg/dL. FDG infusion and PET imaging were initiated upon achievement of steady state glucose infusion, at 98±3 min after the initiation of insulin infusion. The clamp was maintained during the PET imaging, with the glucose infusion rate averaging 26±0.4 mg/kg/min and the blood glucose averaging 90±1.7 mg/dL.

### Glucose infusions with sampling

Glucose was infused via a double-lumen left-sided uterine artery catheter for 48 hours on gestational days 18-20. The double-lumen consisted of two adjacent heat-stretched, heat-melded PE10 catheters. Dextrose in 0.9% saline with 5 U/mL heparin was infused via the upstream catheter tip at a rate of 4 mg/min and 20 µl/min. The second lumen tip, downstream by 2.5 mm, was used for blood sampling and kept patent by 0.9% saline with 5 units/mL heparin infused at 10 µL/min. Simultaneous maternal blood was obtained by tail-nick.

### Statistical analyses

Error bars represent standard error of the means. Comparisons between two independent samples were performed using two-tailed homoscedastic Student's *t*-tests. Paired samples were compared using two-tailed repeated measures *t*-tests.

## Supporting Information

Figure S1 Plasma sampling during PET. (a) Average serum ^18^F concentration as sampled from the tail or right femoral artery, showing correlation (b) from samples collected from both sites simultaneously (*r* = 0.80, *p*<10^-23^, N = 104). (c) Serum glucose as measured from the tail or right femoral artery, showing correlation (d) from samples collected from both sites simultaneously (*r* = 0.83, *p*<10^-24^, N = 92). (e) Average serum ^3^H concentration as sampled from the tail or right femoral artery, showing correlation (f) from samples collected from both sites simultaneously (*r* = 0.87, *p*<10^-28^, N = 95).
(0.44 MB TIF)

Figure S2 Fetal and placental weights by uterine horn position. Fetal (a) and placental (b) weights are shown by uterine horn position. Position 1 is closest to the uterine cervix, with further positions closer to the ovarian end of the uterine horn.
(0.13 MB TIF)

Figure S3 Impact of early FDG pattern on differential glucose exposure. Dynamic imaging, displayed as maximum intensity projections, shows early FDG disposition caudal relative to the catheter tip, in a scan that ultimately did not exhibit differential glucose accumulation between uterine horns. Minutes (“m”) after initiation of infusion are listed on each image.
(1.45 MB TIF)

Figure S4 Fetal and placental ^18^F by uterine horn position. Position 1 is closest to the uterine cervix, with further positions closer to the ovarian end of the uterine horn. Per fetus (a) and per placenta (b) accumulation of ^18^F. *p<0.05 for left versus right.
(0.07 MB TIF)

Video S1 Video of representative dynamic PET. Serial dynamic images collected during time 0-90 minutes, shown as maximum intensity projections.
(1.33 MB GIF)

## References

[1] Finnell RH, Waes JG, Eudy JD, Rosenquist TH (2002). Molecular basis of environmentally induced birth defects.. Annu Rev Pharmacol Toxicol.

[2] Maayan-Metzger A, Lubin D, Kuint J (2009). Hypoglycemia rates in the first days of life among term infants born to diabetic mothers.. Neonatology.

[3] Weiss SJ, St Jonn-Seed M, Harris-Muchell C (2007). The contribution of fetal drug exposure to temperament: potential teratogenic effects on neuropsychiatric risk.. J Child Psychol Psychiatry.

[4] Parman T, Wiley MJ, Wells PG (1999). Free radical-mediated oxidative DNA damage in the mechanism of thalidomide teratogenicity.. Nat Med.

[5] Daston GP, Yonker JE, Powers JF, Heitmeyer SA (1989). Difference in teratogenic potency of ethylenethiourea in rats and mice: relative contribution of embryonic and maternal factors.. Teratology.

[6] Khera KS (1987). Maternal toxicity of drugs and metabolic disorders–a possible etiologic factor in the intrauterine death and congenital malformation: a critique on human data.. Crit Rev Toxicol.

[7] Correa A, Gilboa SM, Besser LM, Botto LD, Moore CA (2008). Diabetes mellitus and birth defects.. Am J Obstet Gynecol.

[8] Rasmussen SA, Chu SY, Kim SY, Schmid CH, Lau J (2008). Maternal obesity and risk of neural tube defects: a metaanalysis.. Am J Obstet Gynecol.

[9] Freinkel N (1980). Banting Lecture 1980. Of pregnancy and progeny.. Diabetes.

[10] Schmatz M, Madan J, Marino T, Davis J (2009). Maternal obesity: the interplay between inflammation, mother and fetus.. J Perinatol.

[11] Wells PG, Bhuller Y, Chen CS, Jeng W, Kasapinovic S (2005). Molecular and biochemical mechanisms in teratogenesis involving reactive oxygen species.. Toxicol Appl Pharmacol.

[12] Boney CM, Verma A, Tucker R, Vohr BR (2005). Metabolic syndrome in childhood: association with birth weight, maternal obesity, and gestational diabetes mellitus.. Pediatrics.

[13] Segar EM, Norris AW, Yao J, Hu S, Koppenhafer SL (2009). Programming of growth, insulin resistance and vascular dysfunction in offspring of late gestation diabetic rats.. Clin Sci (Lond).

[14] Buchanan TA, Denno KM, Sipos GF, Sadler TW (1994). Diabetic teratogenesis. In vitro evidence for a multifactorial etiology with little contribution from glucose *per se*.. Diabetes.

[15] Sadler TW, Hunter ES3, Wynn RE, Phillips LS (1989). Evidence for multifactorial origin of diabetes-induced embryopathies.. Diabetes.

[16] Schaefer-Graf UM, Graf K, Kulbacka I, Kjos SL, Dudenhausen J (2008). Maternal lipids as strong determinants of fetal environment and growth in pregnancies with gestational diabetes mellitus.. Diabetes Care.

[17] Gauguier D, Bihoreau MT, Picon L, Ktorza A (1991). Insulin secretion in adult rats after intrauterine exposure to mild hyperglycemia during late gestation.. Diabetes.

[18] Stein DT, Stevenson BE, Chester MW, Basit M, Daniels MB (1997). The insulinotropic potency of fatty acids is influenced profoundly by their chain length and degree of saturation.. J Clin Invest.

[19] Hirano T, Mamo JC, Furukawa S, Nagano S, Takahashi T (1990). Effect of acute hyperglycemia on plasma triglyceride concentration and triglyceride secretion rate in non-fasted rats.. Diabetes Res Clin Pract.

[20] Eriksson UJ (2009). Congenital anomalies in diabetic pregnancy.. Semin Fetal Neonatal Med.

[21] Moore DC, Stanisstreet M, Clarke CA (1989). Morphological and physiological effects of beta-hydroxybutyrate on rat embryos grown *in vitro* at different stages.. Teratology.

[22] Rizzo T, Metzger BE, Burns WJ, Burns K (1991). Correlations between antepartum maternal metabolism and child intelligence.. N Engl J Med.

[23] Styrud J, Thunberg L, Nybacka O, Eriksson UJ (1995). Correlations between maternal metabolism and deranged development in the offspring of normal and diabetic rats.. Pediatr Res.

[24] Massa HM, Bruce NW (1997). Direction of blood flow and changes in resistance of major arteries supplying the ovary of the pregnant rat.. Biol Reprod.

[25] Bertuzzi A, Mingrone G, De Gaetano A, Gandolfi A, Greco AV (1997). Kinetics of dodecanedioic acid and effect of its administration on glucose kinetics in rats.. Br J Nutr.

[26] Bruce N, Parkinson S (1979). Effect of nicotine on uterine blood flow in anesthetized pregnant rats.. Biol Reprod.

[27] Holemans K, Aerts L, Van Assche FA (2003). Fetal growth restriction and consequences for the offspring in animal models.. J Soc Gynecol Investig.

[28] Schneidereit M (1985). Study of fetal organ growth in Wistar rats from day 17 to 21.. Lab Anim.

[29] Even MD, Laughlin MH, Krause GF, vom Saal FS (1994). Differences in blood flow to uterine segments and placentae in relation to sex, intrauterine location and side in pregnant rats.. J Reprod Fertil.

[30] Lasuncion MA, Testar X, Palacin M, Chieri R, Herrera E (1983). Method for the study of metabolite transfer from rat mother to fetus.. Biol Neonate.

[31] Mayer M, Chahoud I, Krowke R, Neubert D (1992). Embryonic and maternal tissue concentration of TCDD in the rat following infusion into the arteria uterina.. Chemosphere.

[32] Richter SH, Garner JP, Auer C, Kunert J, Würbel H (2010). Systematic variation improves reproducibility of animal experiments.. Nat Methods.

[33] Buchanan TA, Leroith D, Taylor SI, Olefsky JM (2000). Effects of maternal diabetes mellitus on intrauterine development.. Diabetes mellitus.

[34] Bihoreau MT, Ktorza A, Kinebanyan MF, Picon L (1986). Impaired glucose homeostasis in adult rats from hyperglycemic mothers.. Diabetes.

[35] Fauque P, Mondon F, Letourneur F, Ripoche M, Journot L (2010). *In vitro* fertilization and embryo culture strongly impact the placental transcriptome in the mouse model.. PLoS One.

[36] Scialli AR, Iannucci AR (2010). Whole embryo culture and the identification of “teratogenicity”.. Reprod Toxicol.

[37] Flake AW, Villa-Troyer RL, Adzick NS, Harrison MR (1986). Transamniotic fetal feeding. III. The effect of nutrient infusion on fetal growth retardation.. J Pediatr Surg.

[38] Zawiejska A, Wender-Ozegowska E, Brazert J, Sodowski K (2008). Components of metabolic syndrome and their impact on fetal growth in women with gestational diabetes mellitus.. J Physiol Pharmacol.

[39] Loeken MR (2008). Challenges in understanding diabetic embryopathy.. Diabetes.

[40] Oakes ND, Kjellstedt A, Forsberg GB, Clementz T, Camejo G (1999). Development and initial evaluation of a novel method for assessing tissue-specific plasma free fatty acid utilization *in vivo* using (R)-2-bromopalmitate tracer.. J Lipid Res.

[41] Fraser I, Pearson H, Bowry V, Bell PR (1984). The intravenous intralipid tolerance test.. J Leukoc Biol.

[42] Barton RN (1976). Effect of ischaemic limb injury on the rates of metabolism of ketone bodies in starved rats... Biochem J.

[43] Green E (1935). Anatomy of the Rat.. Trans Am Philos Soc.

