# Supplementary figures and images for: Localized Fetomaternal Hyperglycemia: Spatial and Kinetic Definition by Positron Emission Tomography
Source: PLoS One. 2010 Aug 6;5(8):e12027. doi: 10.1371/journal.pone.0012027 (PMC2917372; doi:10.1371/journal.pone.0012027)

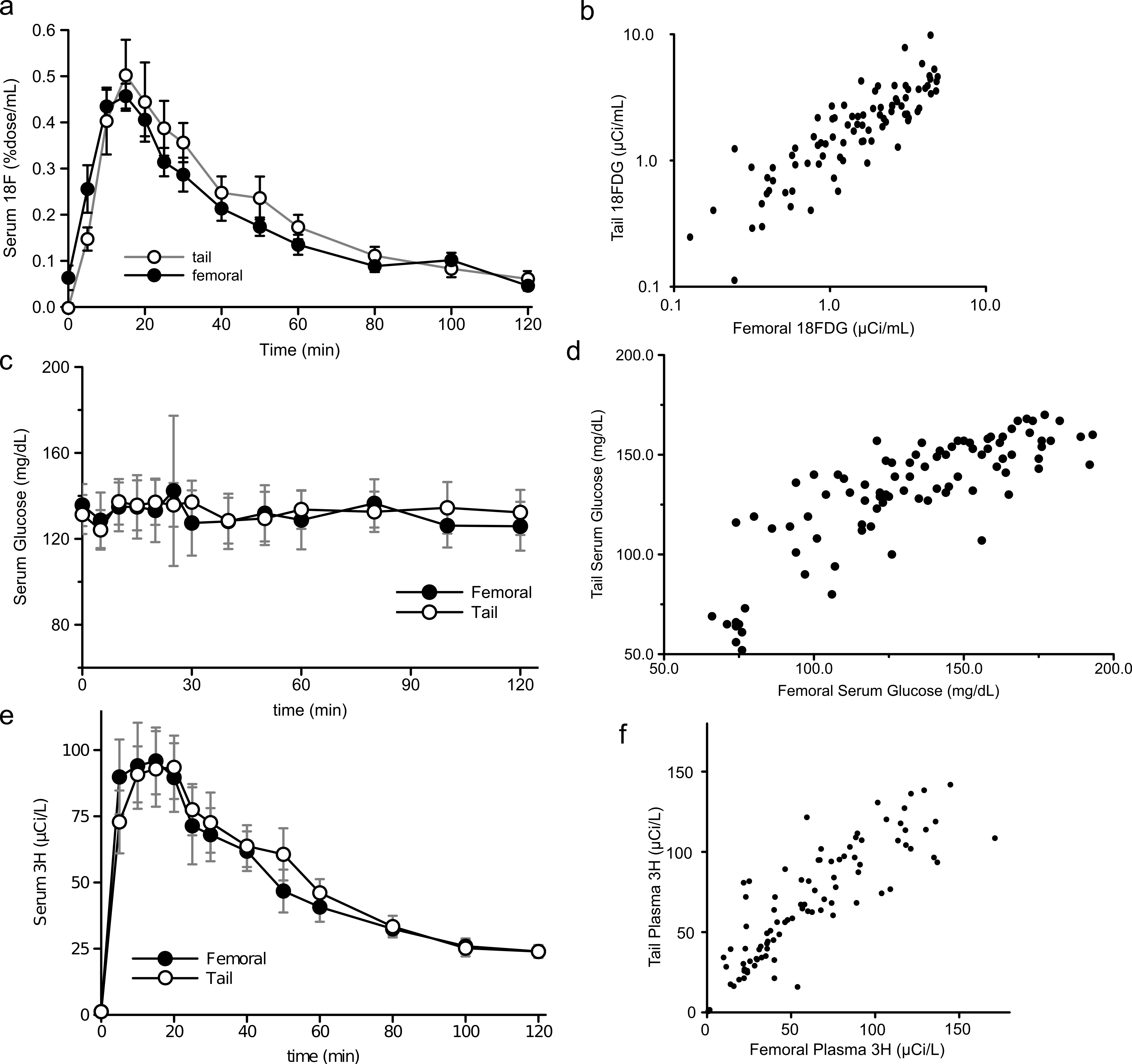

Supplement: Figure S1 — Plasma sampling during PET. (a) Average serum 18F concentration as sampled from the tail or right femoral artery, showing correlation (b) from samples collected from both sites simultaneously (r = 0.80, p<10-23, N = 104). (c) Serum glucose as measured from the tail or right femoral artery, showing correlation (d) from samples collected from both sites simultaneously (r = 0.83, p<10-24, N = 92). (e) Average serum 3H concentration as sampled from the tail or right femoral artery, showing correlation (f) from samples collected from both sites simultaneously (r = 0.87, p<10-28, N = 95). (0.44 MB TIF) [file pone.0012027.s001.tif]

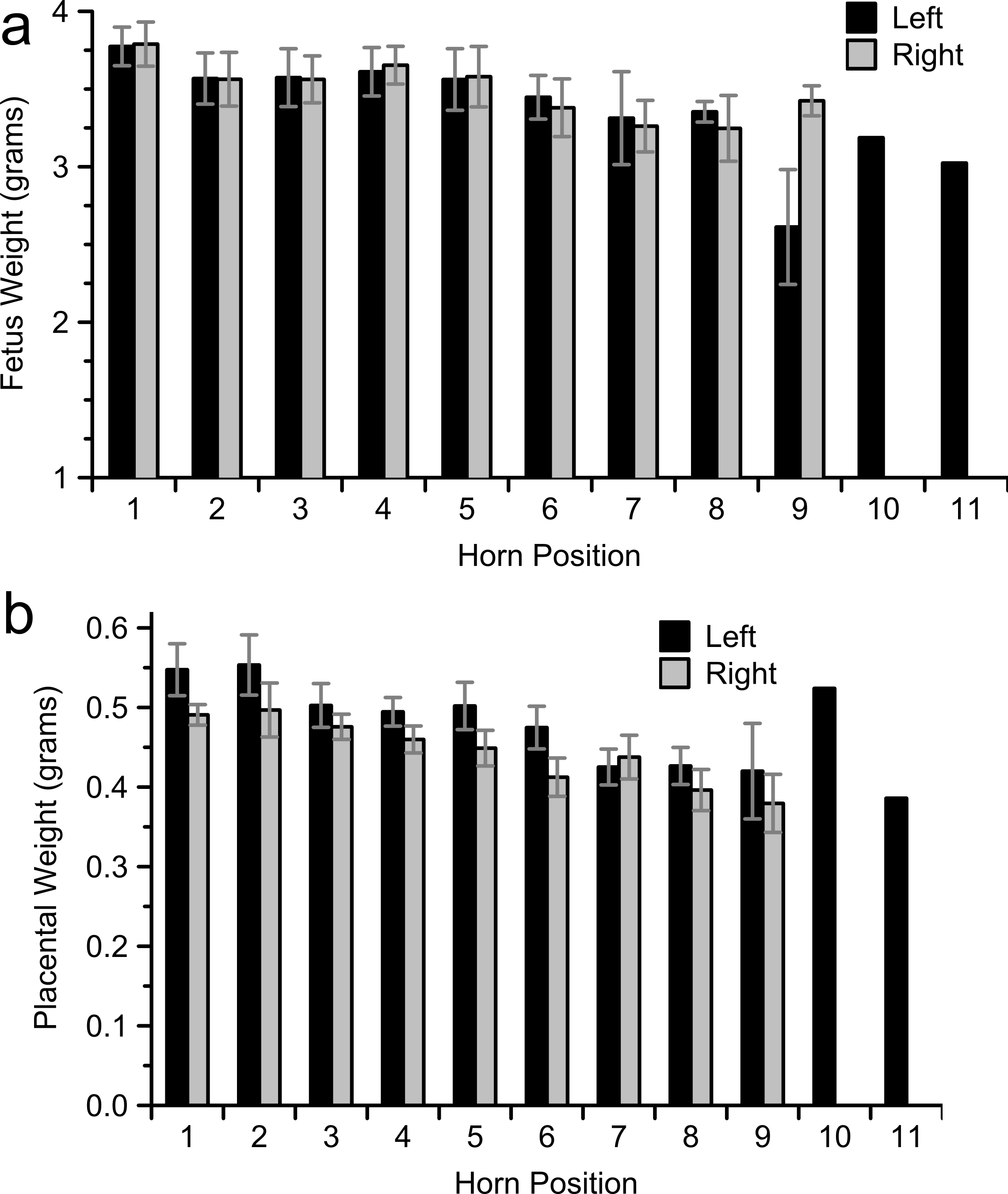

Supplement: Figure S2 — Fetal and placental weights by uterine horn position. Fetal (a) and placental (b) weights are shown by uterine horn position. Position 1 is closest to the uterine cervix, with further positions closer to the ovarian end of the uterine horn. (0.13 MB TIF) [file pone.0012027.s002.tif]

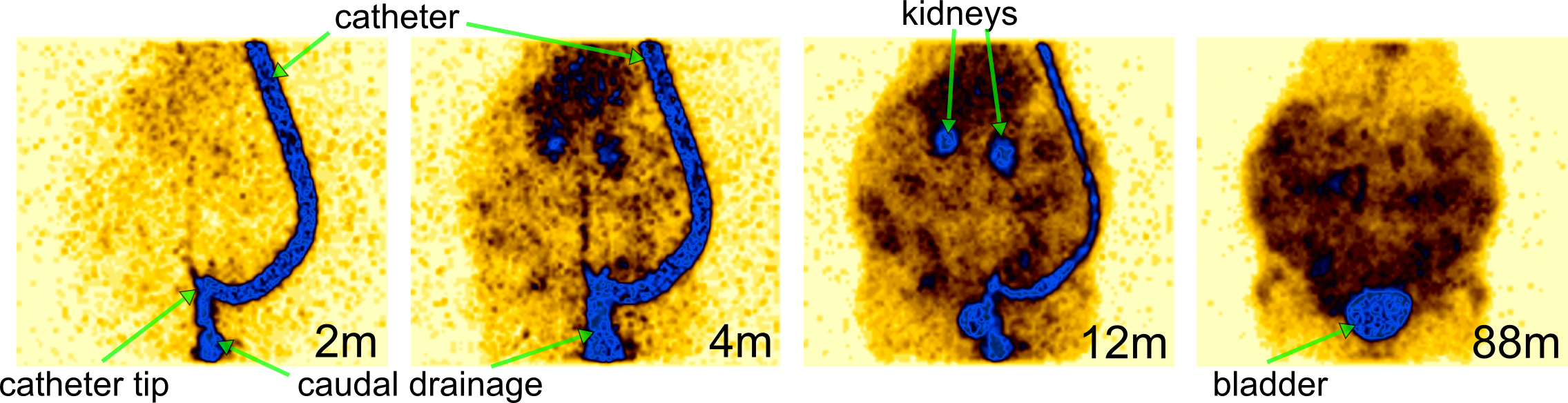

Supplement: Figure S3 — Impact of early FDG pattern on differential glucose exposure. Dynamic imaging, displayed as maximum intensity projections, shows early FDG disposition caudal relative to the catheter tip, in a scan that ultimately did not exhibit differential glucose accumulation between uterine horns. Minutes (“m”) after initiation of infusion are listed on each image. (1.45 MB TIF) [file pone.0012027.s003.tif]

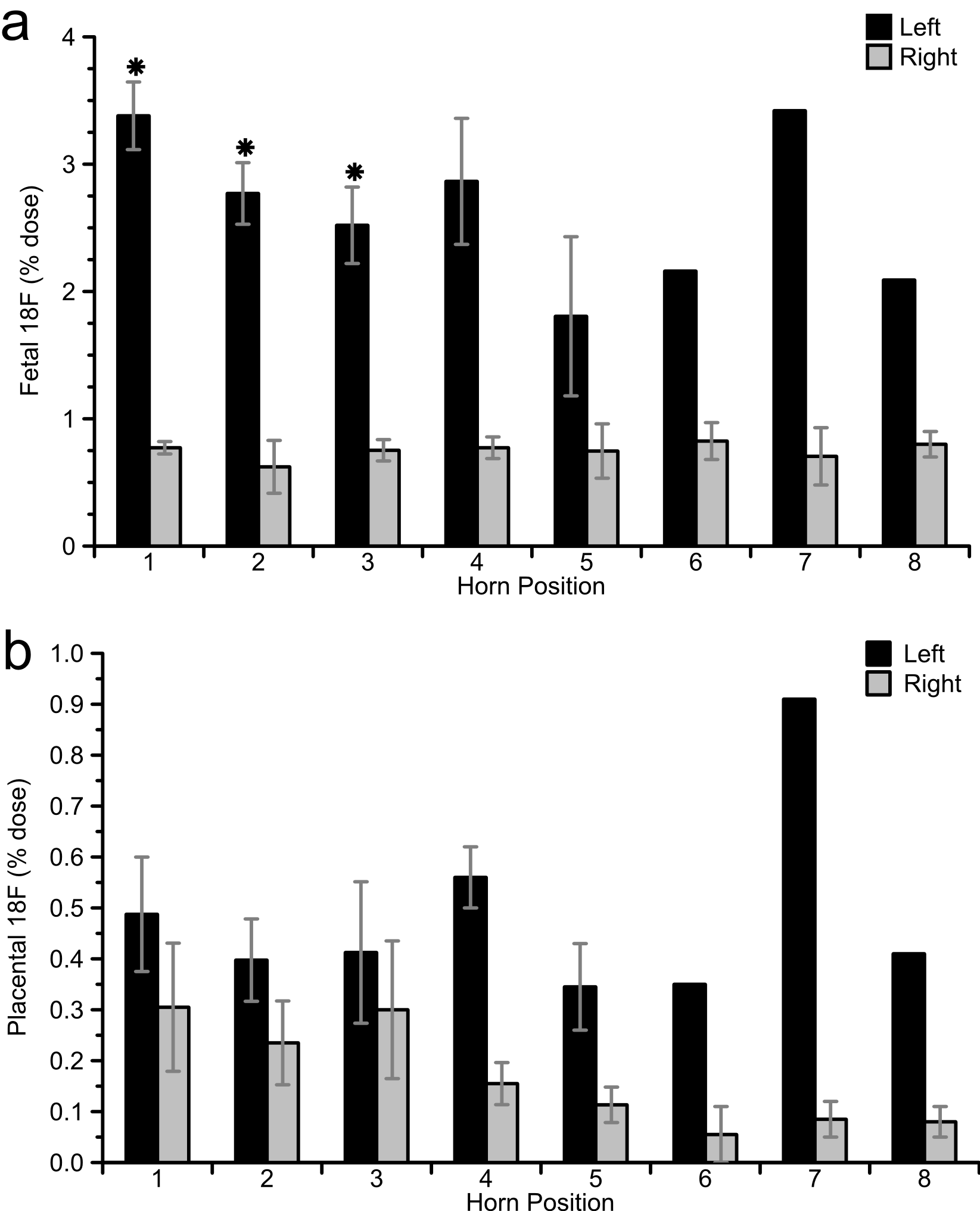

Supplement: Figure S4 — Fetal and placental 18F by uterine horn position. Position 1 is closest to the uterine cervix, with further positions closer to the ovarian end of the uterine horn. Per fetus (a) and per placenta (b) accumulation of 18F. *p<0.05 for left versus right. (0.07 MB TIF) [file pone.0012027.s004.tif]

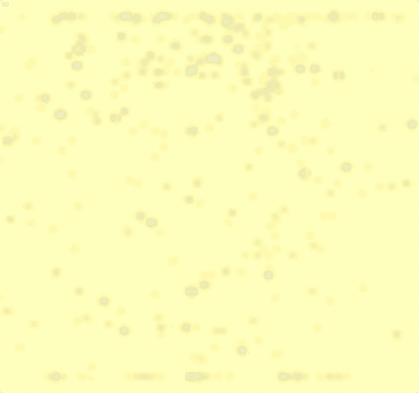

Supplement: Video S1 — Video of representative dynamic PET. Serial dynamic images collected during time 0-90 minutes, shown as maximum intensity projections. (1.33 MB GIF) [file pone.0012027.s005.gif]
